# Current status of short video as a source of information on lung cancer: a cross-sectional content analysis study

**DOI:** 10.3389/fonc.2024.1420976

**Published:** 2024-11-22

**Authors:** Xinyu Zhao, Xinyi Yao, Binbin Sui, Yutao Zhou

**Affiliations:** Department of Respiratory, Liyang People’s Hospital of Jiangsu Province, Liyang, China

**Keywords:** lung cancer, short video, health information, health education, medical knowledge

## Abstract

**Background:**

The morbidity and mortality rates of lung cancer continue to rise, leading to a significant disease burden. Health education on lung cancer serves as an effective approach for prevention and treatment. With the increasing popularity of the Internet, an escalating number of patients are turning to video platforms for health information. Short videos facilitate better absorption and retention of information, thus becoming the primary channel for health education communication. However, the quality of information provided in videos on these platforms remains uncertain. Therefore, this study aims to assess the information quality pertaining to lung cancer in short videos available on a Chinese video platform.

**Methods:**

Lung cancer-related videos on two short video platforms (TikTok and Kwai) were screened, and only Chinese (Mandarin) videos were included. The Global Quality Score (GQS) and modified DISCERN (mDISCERN) tools were then used to evaluate the quality and reliability of the information. A comparative analysis was conducted on videos from various sources. Additionally, correlation analysis was employed to investigate the factors influencing video quality.

**Results:**

After screening, a total of 186 videos were included. The median GQS score and mDISCERN score were 3 (IQR: 3-4) and 2 (IQR: 2-4), respectively. A total of 44.1% of the lung cancer videos provided a comprehensive explanation of the symptoms, while only 3.2% fully explanation the complications associated with lung cancer. Health professionals, particularly specialists, demonstrated higher quality video information compared to individual users (*P*<0.001). The correlation coefficient between GQS score and mDISCERN score was 0.340, showing a significant positive correlation (*P*<0.001). In addition, GQS score was positively correlated with video duration (r=0.177, *P*=0.015)

**Conclusion:**

The information quality of the 186 videos screened by the two platforms in this study was generally unsatisfactory. However, videos provided by experts were deemed relatively reliable, with video duration being closely associated with information quality. Therefore, it is crucial to meticulously screen high-quality and dependable videos on the platform in order to effectively guide lung cancer prevention and treatment.

## Introduction

The prevalence of lung cancer, a highly aggressive disease, exerts a significant impact on the escalating burden of cancer-related mortality worldwide (1–3). In 2020 alone, there were 2.2 million newly diagnosed cases and 1.8 million reported deaths attributed to this condition (1). The latest epidemiological studies conducted in Europe indicate that lung cancer accounts for 20% of all deaths in the region, with a persistently increasing mortality rate observed among the elderly population (2). The trend of cancer burden in China from 2005 to 2020 revealed that trachea, bronchus, and lung cancer had the highest mortality rate among males, reaching 75.5 per 100,000 (3). The incidence and mortality rates of lung cancer exhibit significant variations across different regions worldwide, with countries characterized by higher levels of economic development, as measured by the Human Development Index (HDI), experiencing three to four times greater incidence and mortality rates (1, 4). These variations can be attributed to factors such as tobacco consumption patterns, gender disparities, and divergent economic trends (5).

The mortality rate of lung cancer in developed countries has witnessed a decline in recent years, primarily attributed to the implementation of tobacco control measures, enhanced screening programs, and improved treatment options (1). In low- and middle-income countries, there exist both patient-related barriers and obstacles within national healthcare systems (4). Apart from limited accessibility to state-of-the-art therapies for lung cancer, primary healthcare professionals lack sufficient knowledge regarding the latest screening guidelines (6). Lung cancer screening guidelines mainly use low-dose-computed-tomography (LDCT), followed by bronchoscopy and sputum screening. The latest screening protocols include artificial intelligence-assisted CT examination and liquid biopsy (7). Patients themselves may also exhibit inadequate awareness about lung cancer, a lack of understanding regarding the benefits of early screening, and possess fatalistic views towards this disease (8, 9). Therefore, a comprehensive understanding of the etiology of lung cancer, early screening methods, and standardized treatment can significantly enhance patients’ survival rates.

The reform of information technology is impacting people’s daily life in various ways. It also offers patients effective health communication methods, enabling them to access relevant disease management information and empowering them with disease management capabilities (10). Visual information platforms, such as TikTok and Kwai, offer more visually appealing image and video content that facilitates patients’ comprehension and retention of information. Several studies (11–14) have examined health-related information available on these platforms, including chronic obstructive pulmonary disease (COPD), inflammatory bowel disease (IBD), and cancer. These studies have found that patients are more inclined to accept and remember visually engaging relevant information (15, 16). In addition, there is evidence (17, 18) that active use of the visual information platform is associated with a good prognosis of patients, the platform helps patients with self-disease management, and reduces the psychological burden of patients. However, short video platforms can lead to the rapid spread of false health-related information, which can mislead patients’ disease management decisions and even pose a threat to their lives. Health information on the Internet is often complex and challenging for non-professionals to comprehend, especially for individuals with limited health literacy. A study (19) revealed that over half of COPD patients encountered difficulties in discerning between high- and low-quality health information online. Therefore, it is crucial to assess the credibility of image-based information platforms and guide lung cancer patients towards standardized disease management protocols in order to reduce lung cancer mortality rates.

Previous studies have evaluated the quality of health information of several diseases on TikTok (20–22), but the quality of lung cancer short video information is still unclear. In order to improve the information content of lung cancer short video and guide the disease management of lung cancer patients, this study will examine the quality of lung cancer information on domestic short video platforms and evaluate the quality of lung cancer related health information.

## Methods

### Data collection

Between April 6, 2024 and April 7, 2024, we conducted a cross-sectional study. In this study, the Chinese keyword “lung cancer” was used to search for related videos on the two most popular short video sharing platforms in China (TikTok and Kwai), and the default top 100 videos were screened. Videos that met the following criteria were excluded from the analysis: 1) repetitive videos; 2) silent videos and 3) videos that were unrelated to the topic. The video was screened by two respiratory doctors (Xinyu Zhao and Xinyi Yao), and the content and quality of the video were independently reviewed. The data were recorded in Excel (Microsoft Corporation) and it was agreed that a third senior respiratory specialist (Binbin Sui) would conduct a negotiated assessment of the disputed issues. The following video information was collected: the platform of videos, the source of videos, the identity authentication of the publisher, date of publication, departments of the medical worker, duration of videos, the number of likes, comments and collections, the content and the quality of the videos. The data of this study are publicly available and no ethical statement is required.

### Assessment of video information’s quality and reliability

All searches were conducted on a public computer, all Settings and history on the computer were deleted before the search is conducted, and cookies were disabled during the search process to avoid affecting the data. The Global Quality Score (GQS) and the modified DISCERN (mDISCERN) tool were utilized for assessing the information quality and reliability of videos, respectively. The GQS is a widely used video scoring tool to assess the informational quality of a video and consists mainly of five criteria ranging from 1 (poor quality) to 5 (high quality), with higher scores indicating higher quality, as detailed in **Supplementary Table 1** (23, 24). The reliability of health-related content was evaluated using the mDISCERN tool (11, 25). The instrument consists of five questions (**Supplementary Table 2**) that are scored on the basis of a “yes” or “no” response, with a minimum score of 0 and a maximum score of 5. At the same time, we also assessed the video content and employed an additional scoring tool to evaluate seven key aspects of lung cancer videos, encompassing epidemiology, etiology, symptoms, diagnosis, treatment, prevention, and complications. The scoring system was categorized into three levels: no inclusion (0 points), partial elucidation (1 point), and full explanation (2 points). Specialists include doctors in respiratory, thoracic surgery and oncology, while non-specialists mainly include doctors in other departments such as dermatology and gastroenterology.

### Statistical analysis

Continuous variables were expressed as mean ± standard deviation (SD) or median and interquartile range (IQR) depending on whether they followed a normal distribution, and the Student’s t test or Mann-Whitney U test was used to analyze the data. Categorical variables were presented in frequency and percentage, and Chi-square tests or Fisher exact tests were used. The Pearson correlation coefficient was calculated to evaluate the correlation between different scores and video features. Cohen’s kappa coefficient was used to assess the consistency of the scores of two independent reviewers. Statistical analysis and plotting were performed using SPSS version 26.0 (IBM; Chicago, IL, USA) and R statistical software version 4.3.1 (www.r-project.org). P value < 0.05 was considered to indicate statistical significance.

## Results

### Short video features

**Figure 1:**
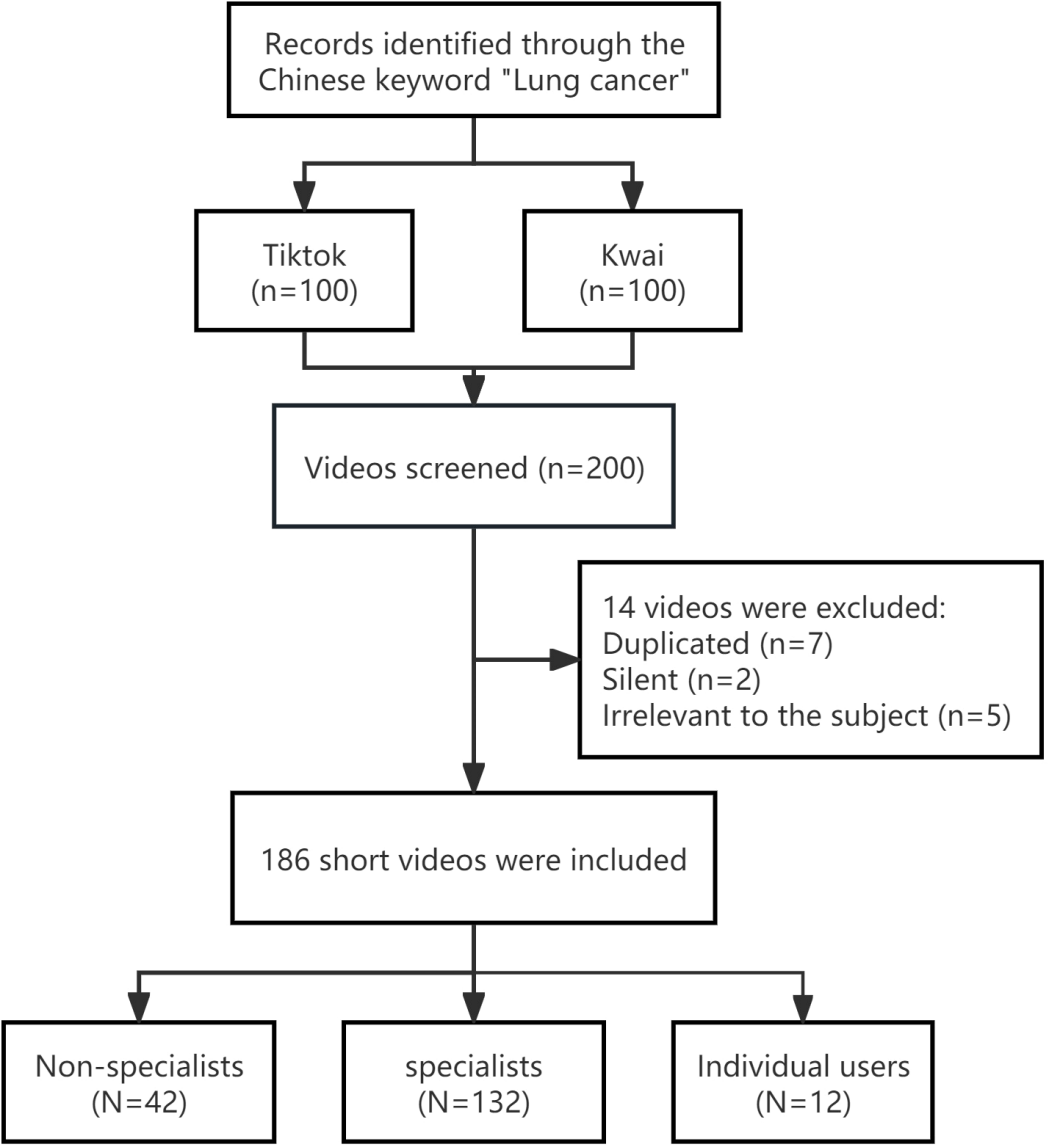
Lung cancer related videos were included in the flow chart.

The video platforms TikTok and Kwai are widely regarded as the two most famous platforms in China. After carefully screening the top 100 comprehensive video messages on two platforms, we eliminated duplicate, silent, and irrelevant content, and ultimately selected 186 short video messages (**Figure 1**). We have classified the sources of videos into three categories: specialists, non-specialists and individual users. Out of these videos, 71% were uploaded by professionals, 22.6% by non-professionals, and 6.4% by individual users. The median video duration was 72 seconds (Interquartile range [IQR]:21-101), the median number of likes and collections was 633(IQR:173-4010) and 132(IQR:28-636), respectively, and the median number of comments received was 58(IQR:13-343). The median completeness score was 6(IQR:5-8), the median GQS was 3(IQR:3-4), and the median mDISCERN score was 2(IQR:2-4) (**Table 1**). The quality assessment revealed that the lung cancer video information on the platform exhibited a low level of quality.

**Table 1:** Characteristics of 186 lung cancer related-short videos on TikTok and Kwai in China in 2024.

Characteristic		N=186
Short-video sharing platforms [n(%)]		
	TikTok	95 (51.1)
	Kwai	91 (48.9)
Video source [n(%)]		
	Non-specialists	42 (22.6)
	Specialists	132 (71.0)
	Individual user	12 (6.4)
Number of likes [median(IQR)]		633 (137-4010)
Number of comments [median(IQR)]		58 (13-343)
Number of collections [median(IQR)]		132 (28-636)
Video duration [s, median(IQR)]		72 (21-101)
Completeness score [median(IQR)]		6 (5-8)
GQS scores [median(IQR)]		3 (3-4)
mDISCERN scores [median(IQR)]		2 (2-4)

N,nubmer; IQR,interquartile range; GQS, Global Quality Score; mDISCERN, modified DISCERN.

### Short video content

**Figure 2:**
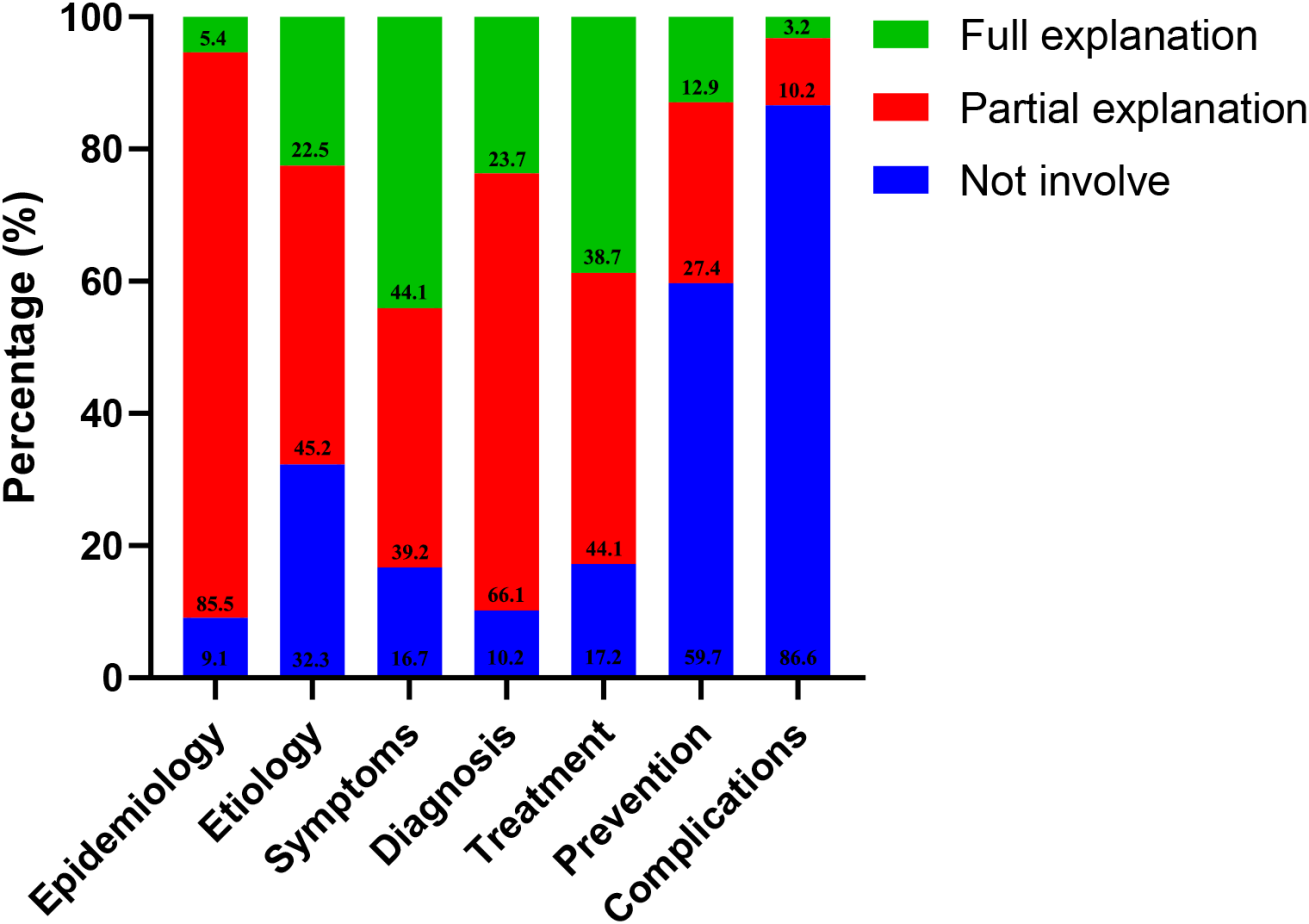
Percentage of videos involving content for each lung cancer.

We classified the completeness of the video content into seven aspects, encompassing epidemiology, etiology, symptoms, diagnosis, treatment, prevention, and complications associated with lung cancer. As shown in **Figure 2**, most of the videos comprehensively described the typical symptoms associated with lung cancer (44.1%), however, only a few videos introduced the complications of lung cancer (3.2%). Moreover, only a subset of the video full explanation the remaining 5 aspects of lung cancer. Among them, a mere 5.4% of the videos provide an in-depth explanation of the epidemiology of lung cancer, while 22.5%, 38.7%, and 12.7% of the videos respectively delve into the etiology, diagnosis, and treatment of this disease. (**Table 2**).

**Table 2:** Completeness of 186 lung cancer related-short videos content on TikTok and Kwai in China in 2024.

Video content	Not involve (0 points)	Partial explanation (1 point)	Full explanation (2 points)
Epidemiology, n (%)	17 (9.1)	159 (85.5)	10 (5.4)
Etiology, n (%)	60 (32.3)	84 (45.2)	42 (22.5)
Symptoms, n (%)	31 (16.7)	73 (39.2)	82 (44.1)
Diagnosis, n (%)	19 (10.2)	123 (66.1)	44 (23.7)
Treatment, n (%)	32 (17.2)	82 (44.1)	72 (38.7)
Prevention, n (%)	111 (59.7)	51 (27.4)	24 (12.9)
Complications, n (%)	161 (86.6)	19 (10.2)	6 (3.2)

n,nubmer; IQR,interquartile range; GQS, Global Quality Score; mDISCERN, modified DISCERN.

### Short video source analysis

**Figure 3:**
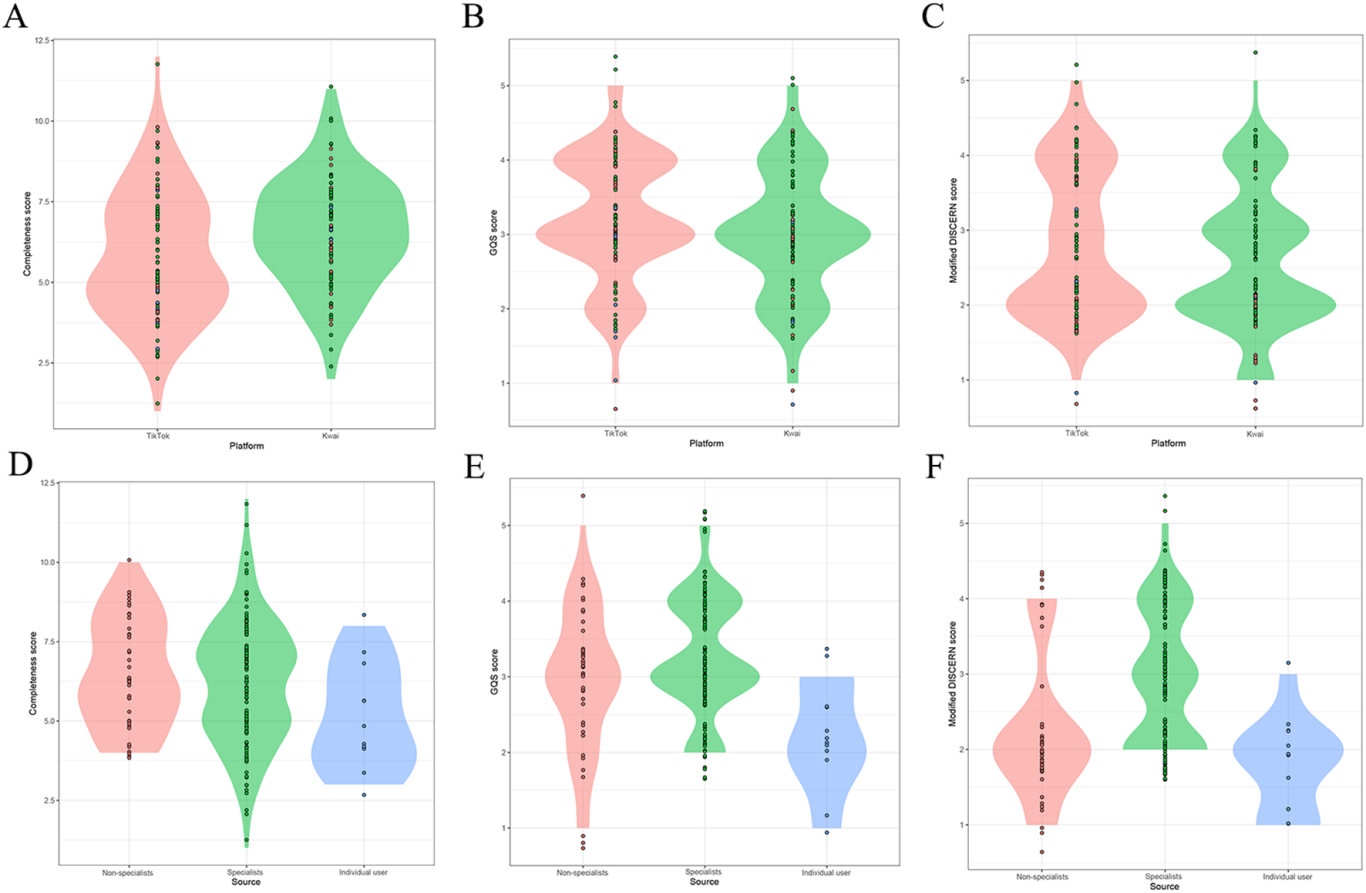
Comparison of video information quality from different sources. **(A–C)** Comparison of completeness score of different platforms, GQS scores and mDISCERN scores by violin plot. **(D–F)** Comparison of completeness score, GQS scores and mDISCERN scores by different users of violin plot.

In addition, we conducted a comparative analysis of the videos on both platforms and observed that TikTok exhibited significantly higher engagement metrics in terms of likes (median: 1706 vs 209, *P*<0.001), comments (median: 173 vs 29, *P*<0.001), and collections (median: 290 vs 49, *P*<0.001) compared to Kwai. Conversely, Kwai demonstrated superior video completeness when compared to TikTok. However, both platforms displayed similar scores in terms of video quality (mDISCERN scores, median:2 vs 2, *P*=0.131) (**Table 3**). We conducted a further analysis of videos from various sources and observed that the quality of video information shared by healthcare professionals surpassed that posted by individual users. Notably, specialists exhibited significantly higher quality scores compared to non-specialists (mDISCERN scores, median: 3 vs 2, *P*<0.001) (**Table 4**). The Cohen’s kappa values for GQS and the mDISCERN were 0.921 and 0.893, respectively, indicating a good agreement between the scores of the two independent reviewers. The correlation between the quality score of the video and its video features is simultaneously examined. As depicted in **Table 5**, the correlation coefficient between GQS score and mDISCERN score was 0.340, showing a significant positive correlation (*P*<0.001). In addition, GQS score was positively correlated with video duration (r=0.177, *P*=0.015). However, the GQS score was not significantly correlated with the number of likes, favorites, and comments. The violin plot, (**Figure 3**), also shows the consistent trend observed. In addition, the information quality scores of the two platforms were not evenly distributed, with a higher proportion of videos with lower scores. It is worth noting that most videos with mDISCERN scores exceeding 3 points were uploaded by specialists.

**Table 3:** Comparison of 186 lung cancer related-short videos in different short-video sharing platforms in China in 2024.

Variables	TikTok (N=95)	Kwai (N=91)	p valve
Video source [n(%)]			0.118
Non-specialists	17 (66.7)	25 (62.5)	
Specialists	75 (14.3)	57 (27.3)	
Individual user	8 (4.7)	4 (1.1)	
Video duration [s, median(IQR)]	76(53-100)	72(48-101)	0.344
Number of likes [median(IQR)]	1706 (696-6526)	209 (56-943)	<0.001
Number of comments [median(IQR)]	173 (32-778)	29 (6-118)	<0.001
Number of collections [median(IQR)]	290 (84-1486)	49 (10-182)	<0.001
Completeness score [median(IQR)]	5 (4-7)	7 (5-8)	0.005
GQS scores [median(IQR)]	3 (3-4)	3 (2-4)	0.064
mDISCERN scores [median(IQR)]	2 (2-4)	2 (2-3)	0.131

n,nubmer; IQR,interquartile range; GQS, Global Quality Score; mDISCERN, modified DISCERN.

**Table 4:** Comparison of 186 lung cancer related-short video in different video source in China in 2024.

Variables	Non-specialists (N=71)	Specialists (N=148)	Individual user (N=9)	p valve
Number of likes [median(IQR)]	594 (107-4172)	667 (137-3778)	984 (156-10052)	0.789
Number of shares [median(IQR)]	45 (10-276)	58 (13-334)	88 (20-1350)	0.680
Number of collections [median(IQR)]	164 (23-1374)	120 (25-560)	194 (40-611)	0.850
Completeness score [median(IQR)]	6 (5-8)	6 (5-7)	4.5 (4-7)	0.073
GQS scores [median(IQR)]	3 (2-4)	3 (3-4)	2 (2-3)	<0.001
mDISCERN scores [median(IQR)]	2 (2-2)	3 (2-4)	2 (1-2)	<0.001

N,nubmer; IQR,interquartile range; GQS, Global Quality Score; mDISCERN, modified DISCERN.

**Table 5:** Correlation analysis between video quality score and video features.

	GQS		mDISCERN	
	r	p valve	r	p valve
GQS	–	–	0.340	<0.001
mDISCERN	0.340	<0.001	–	–
Likes	-0.125	0.088	-0.045	0.545
Comments	-0.068	0.358	-0.061	0.408
Collections	-0.035	0.632	-0.029	0.697
Video duration	0.177	0.015	0.017	0.821

GQS, Global Quality Score; mDISCERN, modified DISCERN.

## Discussion

The visual social platform has gained popularity and demonstrated its advantages in disseminating health knowledge (15, 16). Video platforms replace traditional text-based information with visually appealing content, facilitating easier processing and retention of information for individuals (12, 26, 27). Moreover, the incorporation of health-related content evokes emotional responses and motivates individuals to engage in proactive health behaviors (28). As a highly prevalent and fatal malignant tumor in China, lung cancer imposes a substantial disease burden on the country’s healthcare resources (5). The mortality rate associated with lung cancer continues to rise, particularly in rural areas characterized by low health literacy levels (29, 30), and short video platforms are the most suitable tools for health communication and education. The assurance of video information quality remains a primary concern for these platforms, given the varying levels of information accuracy and potential dissemination of biased or misleading content.

The information quality of the 186 videos screened by the two platforms in this study was generally unsatisfactory. While most videos provided comprehensive presentations on symptoms of lung cancer, there was a lack of coverage on the epidemiology, etiology, diagnosis, and treatment of lung cancer, particularly regarding complications. Notably, almost half of the videos failed to mention any lung cancer-related complications. One potential explanation is that individuals may excessively focus on the direct respiratory symptoms associated with lung cancer, while overlooking the potential impact of lung cancer on other bodily systems, such as the digestive system, endocrine system, and circulatory system. 40.3% of the videos mentioned the prevention of lung cancer, including screening programs such as CT and bronchoscopy. 38.7% of the videos had a detailed explanation of the treatment of lung cancer, and only 17.2% of the videos did not mention the treatment of lung cancer. However, none of the videos mentioned post-chemotherapy/radiotherapy and post- surgical care, which may need to be mentioned in future popular science videos.

In the process of video analysis, we observed distinct disparities in the characteristics between videos on the TikTok and Kwai platforms. Interestingly, TikTok videos exhibited significantly higher engagement metrics such as likes, shares, and comments compared to those on Kwai platform. Moreover, there was a discernible discrepancy in terms of video completeness favoring Kwai over TikTok. This disparity may be attributed to TikTok’s predominant popularity among younger demographics resulting in a relatively lower emphasis on video quality but higher transmission rates. The comparison of video information from various user sources revealed that more than half of the videos were contributed by experts, indicating a higher reliability in video information compared to non-experts and individual users. These findings align with previous studies conducted on the YouTube platform (31, 32). However, it is worth noting that certain videos exhibit subpar information quality. Consequently, we recommend implementing content screening measures and enhancing the informational integrity of health-related videos on this platform to ensure effective lung cancer prevention and management while reducing its incidence and mortality rates. The correlation analysis revealed that there was no significant association between the quality of the video and the number of likes, favorites, and comments from the audience, which contradicts previous research (33, 34) expectations. However, a positive correlation was observed between the duration of the video and its information quality, suggesting that high-quality lung cancer videos tend to offer detailed and comprehensive content rather than being short-lived or focused on generating quick traffic.

Our study has several limitations. Firstly, our evaluation of the quality of lung cancer video information is restricted to the Chinese platform, thus limiting its generalizability to other languages. Secondly, due to the time-sensitive nature of video content, we solely analyzed the quality of video information within a specific timeframe. In addition, we only evaluated the top 100 videos on both platforms, which may not be a comprehensive reflection of the video quality of the platforms. Lastly, our analysis is confined to the two most prominent video platforms in China, future studies should encompass additional platforms for a comprehensive analysis.

## Conclusion

A total of 186 lung cancer videos from both platforms were analyzed in this study. However, the video quality and reliability are suboptimal, and there is a dearth of reports on topics pertaining to lung cancer complications. Nevertheless, videos uploaded by experts elucidating lung cancer exhibited superior quality and comprehensiveness. In the future, it is imperative to further enhance the quality of short video information related to lung cancer and subject it to meticulous expert review for the development of more high-quality content. This will ensure accurate dissemination of knowledge about lung cancer to the platform audience and fortify its prevention and management.

## Data Availability

The original contributions presented in the study are included in the article/**Supplementary Material**, further inquiries can be directed to the corresponding authors.
