# Supplementary material for: Current status of short video as a source of information on lung cancer: a cross-sectional content analysis study
Source: Front Oncol. 2024 Nov 22;14:1420976. doi: 10.3389/fonc.2024.1420976 (PMC11621006; doi:10.3389/fonc.2024.1420976)
Supplement: Supplementary file 1 [file Table1.docx]

**Supplementary Table 1. Global Quality Score (GQS) for assessing the reliability and quality of video. (Scoring ranges from 1 to 5)**

| **GQS Definition** | **Score** |
| --- | --- |
| Poor video quality: Specifically, the content is illogical, poorly flowing, with most information missing and useless to patients | 1 |
| Poor quality in general: the specific performance is realized as follows: poor logic of the content, although some information is listed, but still missing more important information, which is of very limited use to patients | 2 |
| Moderate quality: this is demonstrated by the fact that there is some logic and some important information is fully discussed | 3 |
| Good quality: specifically, the video is logical and smooth, with most relevant information covered and useful to patients | 4 |
| Excellent quality: specifically, the video is logical and the content is very smooth and very useful for patients | 5 |
