# Supplementary material for: Current status of short video as a source of information on lung cancer: a cross-sectional content analysis study
Source: Front Oncol. 2024 Nov 22;14:1420976. doi: 10.3389/fonc.2024.1420976 (PMC11621006; doi:10.3389/fonc.2024.1420976)
Supplement: Supplementary file 2 [file Table2.docx]

**Supplementary Table 2. Modified quality standards of discrimination (scored by answering the following questions, 1 point for "yes", 0 points for "no")**

| **Reliability Score** |
| --- |
| 1. Is the video clear, concise and easy to understand? |
| 2. Is the content presented balanced and unbiased? |
| 3. Does the video have a valid citation? |
| 4. Are there other sources of content listed? |
| 5. Whether the unknown areas of the direction are not mentioned? |
